# Noradrenergic α_1_ Receptor Antagonist Treatment Attenuates Positive Subjective Effects of Cocaine in Humans: A Randomized Trial

**DOI:** 10.1371/journal.pone.0030854

**Published:** 2012-02-03

**Authors:** Thomas F. Newton, Richard De La Garza, Gregory Brown, Thomas R. Kosten, James J. Mahoney, Colin N. Haile

**Affiliations:** Menninger Department of Psychiatry & Behavioral Sciences, Baylor College of Medicine, Michael E. DeBakey Veterans Affairs Medical Center, Houston, Texas, United States of America; Centre for Addiction and Mental Health, Canada

## Abstract

**Background:**

Preclinical research implicates dopaminergic and noradrenergic mechanisms in mediating the reinforcing effects of drugs of abuse, including cocaine. The objective of this study was to evaluate the impact of treatment with the noradrenergic α_1_ receptor antagonist doxazosin on the positive subjective effects of cocaine.

**Methods:**

Thirteen non-treatment seeking, cocaine-dependent volunteers completed this single-site, randomized, placebo-controlled, within-subjects study. In one study phase volunteers received placebo and in the other they received doxazosin, with the order counterbalanced across participants. Study medication was masked by over-encapsulating doxazosin tablets and matched placebo lactose served as the control. Study medication treatment was initiated at 1 mg doxazosin or equivalent number of placebo capsules PO/day and increased every three days by 1 mg. After receiving 4 mg doxazosin or equivalent number of placebo capsules participants received masked doses of 20 and 40 mg cocaine IV in that order with placebo saline randomly interspersed to maintain the blind.

**Results:**

Doxazosin treatment was well tolerated and doxazosin alone produced minimal changes in heart rate and blood pressure. During treatment with placebo, cocaine produced dose-dependent increases in subjective effect ratings of “high”, “stimulated”, “like cocaine”, “desire cocaine”, “any drug effect”, and “likely to use cocaine if had access” (p<.001). Doxazosin treatment significantly attenuated the effects of 20 mg cocaine on ratings of “stimulated”, “like cocaine”, and “likely to use cocaine if had access” (p<.05). There were trends for doxazosin to reduce ratings of “stimulated”, “desire cocaine”, and “likely to use cocaine if had access” (p<.10).

**Conclusions:**

Medications that block noradrenergic α_1_ receptors, such as doxazosin, may be useful as treatments for cocaine dependence, and should be evaluated further.

**Trial Registration:**

Clinicaltrials.gov NCT01062945

## Introduction

A great deal of research aimed at developing treatments for cocaine dependence has focused on agents that directly or indirectly alter functioning of dopaminergic systems, as dopamine (DA) is known to play an important role in mediating cocaine's reinforcing effects [1]. Progress has been limited, however, as DA antagonists are aversive and non-specifically disrupt behavior [2]. Most direct DA agonists have proven ineffective as treatments for cocaine dependence [3], and indirect DA agonists such as amphetamine or methamphetamine, while effective for reducing cocaine use [4], [5], have substantial abuse liability that limits their utility [6].

Genetic and pharmacological evidence has implicated noradrenergic mechanisms in mediating the effects of cocaine and other stimulants [7]. For example, animals which do not express the noradrenergic α_1_ receptor (α_1_R) are insensitive to the locomotor activating effects of cocaine and amphetamine [8], [9], and treatment with the noradrenergic α_1_R antagonist prazosin blocks both cocaine-induced locomotor activation [10], [11] and cocaine-induced reinstatement of extinguished cocaine self-administration in rats [12].

Prazosin is the prototypical α_1_R antagonist. Prazosin has an elimination half-life of 2-3 hours in humans [13], and this limits its potential clinical utility because most patients cannot reliably adhere to dosing regimens that require dosing throughout the day. Doxazosin is a newer α_1_R antagonist with an elimination half-life of 22 hours in humans [14], allowing once-daily dosing. Although early reports indicated that doxazosin had poor brain penetration [15], [16], the side-effects of doxazosin, which include fatigue, dizziness, and somnolence, suggest that doxazosin acts centrally.

We assessed the impact of doxazosin treatment on cocaine's effects using a double-blind, placebo-controlled, within-subjects design in non-treatment-seeking, cocaine-dependent volunteers. We hypothesized that doxazosin treatment would attenuate the subjective effects of cocaine.

## Materials and Methods

### Participants

Non-treatment-seeking, cocaine-dependent participants were recruited through advertisements and were paid for their participation. They received $50 per day for inpatient components of the study and received a $100 completion bonus. All participants met DMS-IV criteria for cocaine dependence, were between 18 and 55 years old, had a history of using cocaine by the smoked or IV route, and normal laboratory evaluation, ECG, and vital signs. Exclusion criteria included a history of head trauma, epilepsy, dependence on drugs other than cocaine and nicotine, inability to detect effects of cocaine, or the presence of any other axis I psychiatric disorder. Serious medical conditions such as heart disease, AIDS, and asthma were also exclusionary. Concomitant use of psychotropic medications or medications affecting blood pressure was not allowed. This study was approved by the institutional review board of the Baylor College of Medicine and all participants gave informed consent.

### Assessments

Clinical diagnosis was determined using the MINI [17]. Mood was assessed using the Beck Depression Inventory [18]. Heart rate and blood pressure were measured frequently throughout the study and at several time points following cocaine dosing. Subjective effects of cocaine were measured using visual-analogue scales anchored at 0 (no effect) and 100 (most ever). Ratings were obtained for “high”, “any drug effects”, “stimulated”, “good effects”, “like cocaine”, “bad effects”, “anxious”, “desire cocaine”, and “likely to use cocaine if had access”. Subjective effects ratings were obtained prior to cocaine dosing and at 5 min intervals until 55 min after dosing. Heart rate and blood pressure measures were also collected at the same time points.

### Medications

Doxazosin 1 mg tablets were purchased commercially from Green Park Pharmacy, Houston TX. Doxazosin was over-encapsulated to mask study medication treatment and encapsulated placebo lactose served as the control. Sterile cocaine HCl for human use was provided by NIDA's medication supply program by RTI International, Research Triangle Park, NC. Sterile saline was used to dilute the cocaine to the desired concentration and also served as the placebo for cocaine.

### Study Design

The protocol for this trial and supporting CONSORT checklist are available as supporting information; see Checklist S1 and Protocol S1. The study employed a within-subjects, double-blind, placebo-controlled design. The order in which participants received doxazosin and placebo was counterbalanced across participants. At least 2 weeks separated study episodes to allow medication wash-out. After screening to assure that participants met inclusion and exclusion criteria, participants were enrolled and housed on the Research Commons of the Michael E. DeBakey VA Medical Center. Participants were monitored closely while inpatient and intermittent UAs were performed to ensure that participants remained abstinent for substances not administered as part of the protocol. Participants were discharged from the hospital between the study episodes. Study medication was started at one capsule per day in the morning and increased by one capsule every three days until participants reached four capsules per day. Resting and orthostatic blood pressure measurements were monitored frequently, as doxazosin can cause hypotension, especially after increases in the dose.

### Cocaine Dosing

When participants reached 4 mg doxazosin/placebo/d (Day 10 of study medication treatment), participants received cocaine (20 and 40 mg, IV) in ascending order with a dose of saline randomly interspersed to maintain the blind. Two of the three doses were given in the morning, separated by 1 h. The third dose was given in the afternoon, several hours after the previous dose. The three doses were sufficiently separated in time to allow effects to completely dissipate between doses [19]. Participants were monitored and discharged from the hospital when stable, generally after 6–8 h.

### Outcomes

The primary outcome was the impact of doxazosin treatment on the cardiovascular and subjective effects of cocaine. We also assessed the tolerability of doxazosin treatment alone in this population, with particular attention to effects of doxazosin treatment on blood pressure. We planned on enrolling 15 participants with the goal of obtaining at least 10 completers.

### Statistical Analysis

For subjective effects measures, the area under to time-effect curve (AUC) was calculated using the trapezoidal rule [20]. For both morning cocaine doses, ratings collected 15 min prior to the first cocaine dose were used as baseline. For the afternoon dose, ratings collected 15 min prior to the afternoon cocaine dose were used as baseline. A two-way repeated measures analysis of variance (ANOVA) was used to analyze effects of doxazosin (0 and 4 mg) on the subjective effects measured following cocaine at three dose levels (0, 20, 40 mg). When main effects were significant, we conducted pairwise multiple comparisons using the Holm-Sidak method so long as the overall significance level was p≤.05. For cardiovascular measures following cocaine dosing, repeated measures ANOVA were calculated, with time (pre-dose to 55 min after cocaine dose) being the repeated measure. We used a repeated measures analysis for cardiovascular measures rather than using the AUC approach because the peak values (e.g. of heart rate and blood pressure) reflect the maximum physiological effects of cocaine and we wanted to document how doxazosin impacted this. We also measured change in cardiovascular measures following study medication dosing at 8 am by comparing cardiovascular measures taken at 9 am to those taken prior to dosing at 7:30 am using a repeated measures ANOVA. The data were tested for normality using the Shapiro-Wilk normality test, and for equal variance using the Levene median test. Statistics were calculated using Sigmaplot version 12 (Systat Software, Inc, San Jose, CA).

## Results

**Figure 1:**
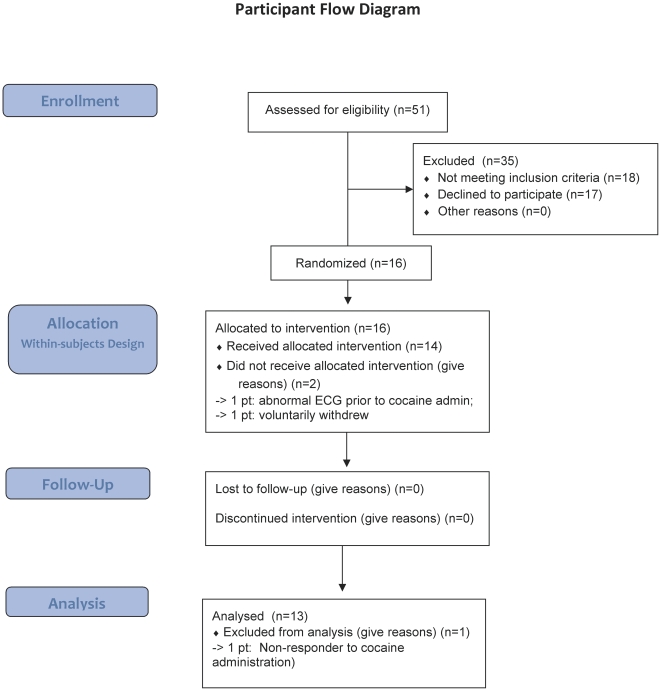
CONSORT Flowchart. Participant flowchart.

The numbers of participants screened, randomized, and the sample analyzed are shown in Figure 1. Participants' demographic and drug use characteristics are shown in Table 1. Participants were middle-aged and used cocaine on average for more than a decade. Most used alcohol and marijuana frequently, though none met criteria for dependence. Most smoked cigarettes and met criteria for nicotine dependence.

**Table 1:** Demographic Characteristics.

	Cocaine Users
	(N = 13)
**Gender (N)**	
Male	12 (92%)
Female	1 (8%)
**Ethnicity (N)**	
Caucasian	3 (23%)
Hispanic	1 (8%)
African American	9 (69%)
**Age (yrs)**	44.31±4.63
**Education (yrs)**	12.46±1.81
**Nicotine Use**	10 (77%)
**Cocaine Use**	
Years of use	18.23±5.55
Number of days used last in last 30 days	15.85±7.87
Grams/day	1.30±0.76
**Route of Admin**	
Smoked	13 (100%)

Data in tables reflect mean ± S. D.

Doxazosin treatment was well tolerated and no participant was discontinued from the study due to side-effects. No participant spontaneously reported sedation, a known side effect of doxazosin, though we did not rate sedation or query participants. Heart rate and blood pressure were not significantly affected. Heart rate and blood pressure measures on the final day of treatment with each doxazosin dose are shown in Table 2. There were no significant differences between measures taken during placebo and doxazosin treatment (p>.10). Change in heart rate and blood pressure following study medication dosing on the first day of treatment with each dose of study medication, when the largest effects would be expected, are shown in Table 3. Doxazosin and placebo both produced minimal changes in blood pressure measured following study medication dosing on the first day of treatment with each dose of study medication (p>.10).

**Table 2:** Heart rate and blood pressure during treatment with doxazosin.

	Baseline	1 mg	2 mg	3 mg	4 mg
Heart Rate	70.15±12.37	75.39±13.05	79.38±10.28	83.08±14.87	83.08±10.85
Systolic BP	121.92±9.31	124.39±7.18	118.39±11.91	121.92±10.14	124.17±12.21
Diastolic BL	79.54±7.51	76.92±8.08	73.84±9.79	77.69±9.07	72.08±9.86

Cardiovascular measures taken following 3 days of treatment with 1, 2, 3 mg/d of doxazosin and following 1 day of treatment with 4 mg doxazosin. None of the values differed from baseline (p>.05).

**Table 3:** Change in heart rate and blood pressure following dosing.

Doxazosin	1 mg	2 mg	3 mg	4 mg
Heart Rate	.61±10.1	−2.92±13.6	6.08±12.01	5.08±9.81
Systolic BP	−.15±8.25	−1.46±8.38	−2.15±9.22	−.46±8.40
Diastolic BL	−1.38±4.33	.69±8.62	.85±12.16	−1.15±10.53

Placebo				
Heart Rate	.23±9.43	2.54±15.40	1.61±13.20	−2.15±9.71
Systolic BP	−.46±1035	1.15±8.38	−1.38±8.93	−2.31±12.24
Diastolic BL	−1.08±7.67	−1.85±5.86	−2.69±6.72	−1.38±8.98

Study medication was dosed at 8am and values represent change (9am minus 7:30am) in heart rate and blood pressure. Measures recorded during treatment with doxazosin did not differ from those recorded during treatment with placebo and no dose differed from another dose (p>.10).

**Figure 2:**
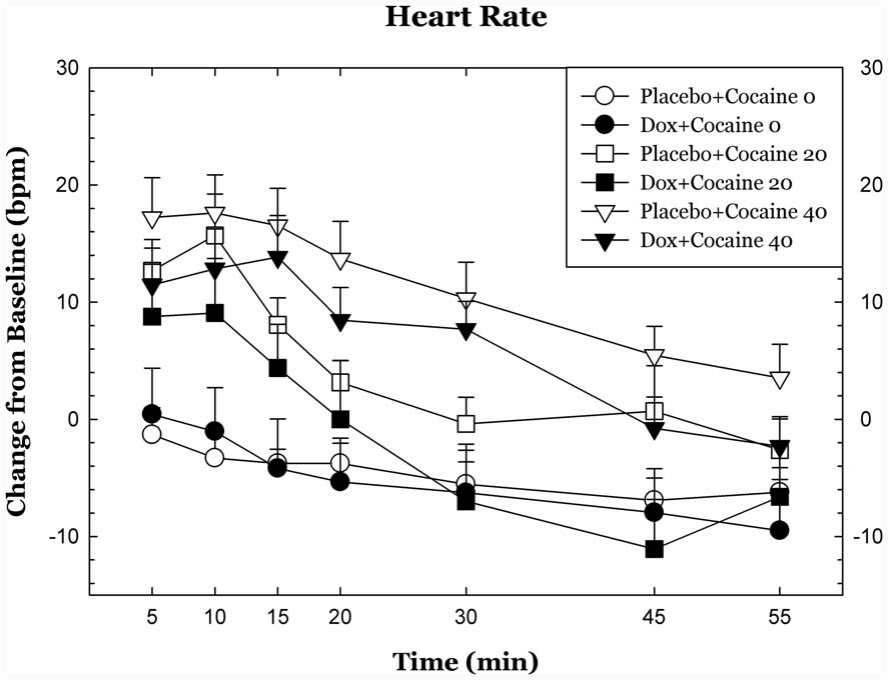
Heart rate and blood pressure following cocaine dosing. Cardiovascular measures are plotted as change from baseline following cocaine administration. There were modest, trend-level reductions in systolic blood pressure effects of cocaine during doxazosin treatment. * p<.05, + p<.10.

**Figure 3:**
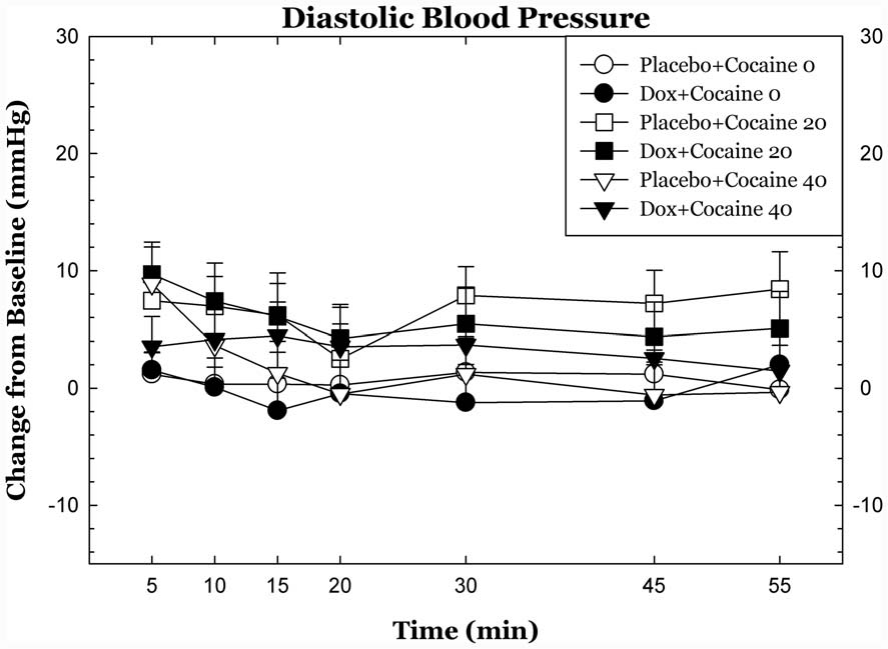
Heart rate and blood pressure following cocaine dosing. Cardiovascular measures are plotted as change from baseline following cocaine administration. There were modest, trend-level reductions in systolic blood pressure effects of cocaine during doxazosin treatment. * p<.05, + p<.10.

**Figure 4:**
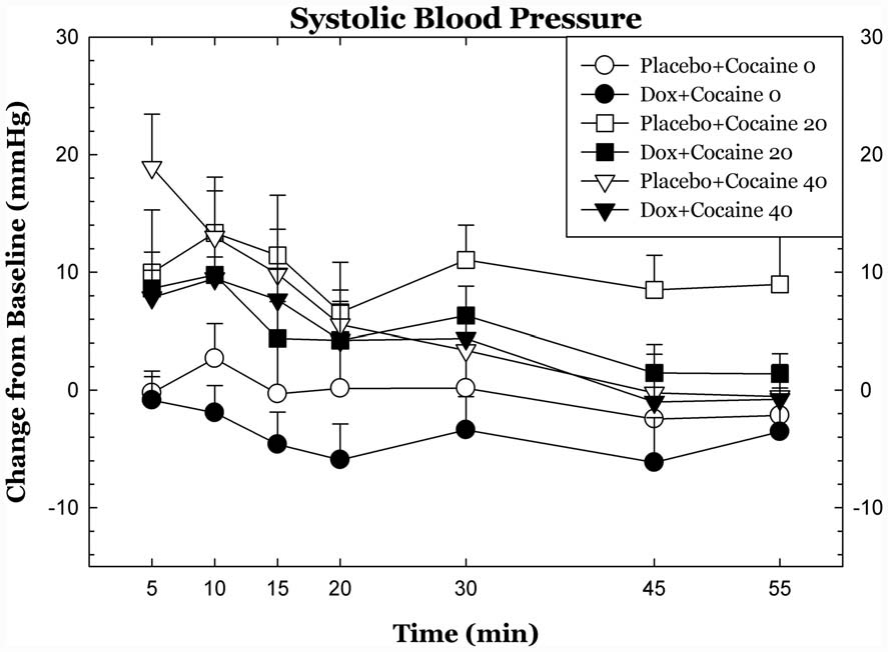
Heart rate and blood pressure following cocaine dosing. Cardiovascular measures are plotted as change from baseline following cocaine administration. There were modest, trend-level reductions in systolic blood pressure effects of cocaine during doxazosin treatment. * p<.05, + p<.10.

Doxazosin had trend-level effects on systolic blood pressure following cocaine dosing (p<.09) but did not significantly affect diasystolic blood pressure or heart rate and there were no statistically significant interactions between cocaine dose and cardiovascular measures. Cardiovascular measures over the 55 min following cocaine dosing are shown in Figures 2, 3, 4.

**Figure 5:**
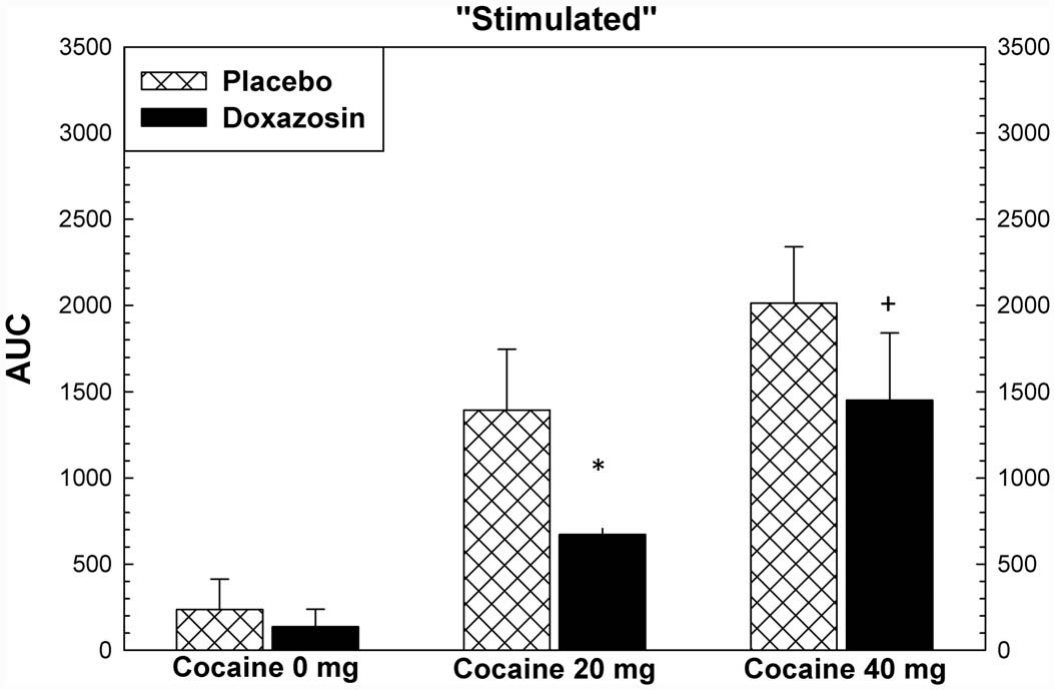
Subjective effects following cocaine dosing. Subjective effects ratings were collected using Likert scales ranging from 0 to 100, with 0 anchored as “not effects” and 100 anchored as “most ever”. ANOVA showed that there were significant effects (p<.05) of medication on ratings of “Stimulated”, “Like Cocaine”, “Desire Cocaine”, and “Likely to Use Cocaine”. Post hoc analysis showed that the statistically significant effects were observed following 20 mg cocaine with trend-level effects following 40 mg cocaine. * p<.05, + p<.10.

**Figure 6:**
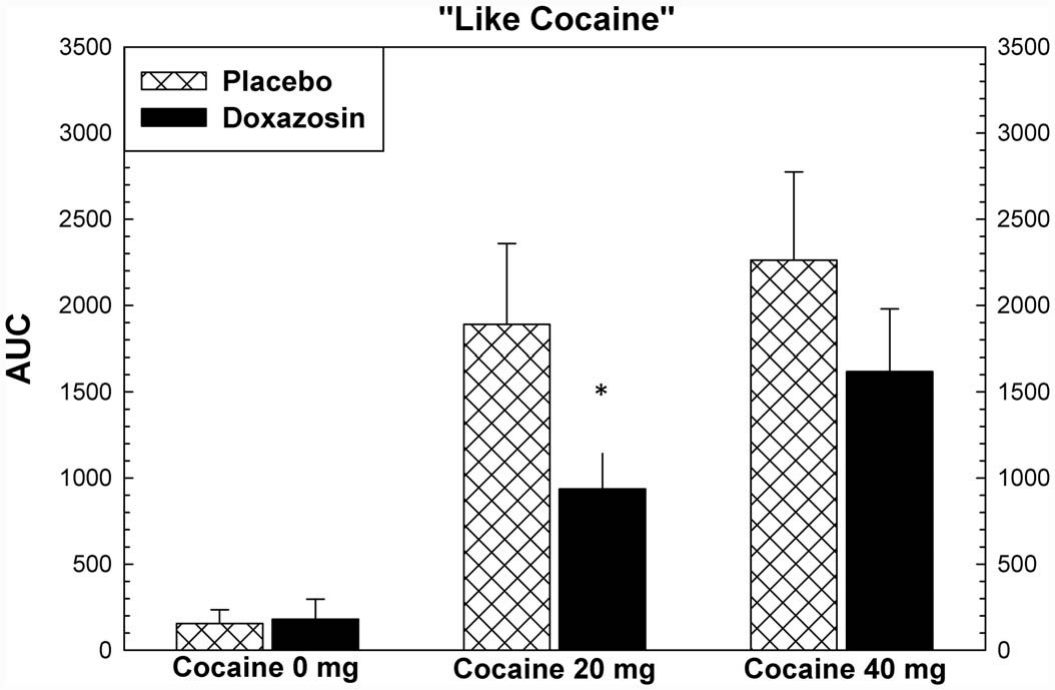
Subjective effects following cocaine dosing. Subjective effects ratings were collected using Likert scales ranging from 0 to 100, with 0 anchored as “not effects” and 100 anchored as “most ever”. ANOVA showed that there were significant effects (p<.05) of medication on ratings of “Stimulated”, “Like Cocaine”, “Desire Cocaine”, and “Likely to Use Cocaine”. Post hoc analysis showed that the statistically significant effects were observed following 20 mg cocaine with trend-level effects following 40 mg cocaine. * p<.05, + p<.10.

**Figure 7:**
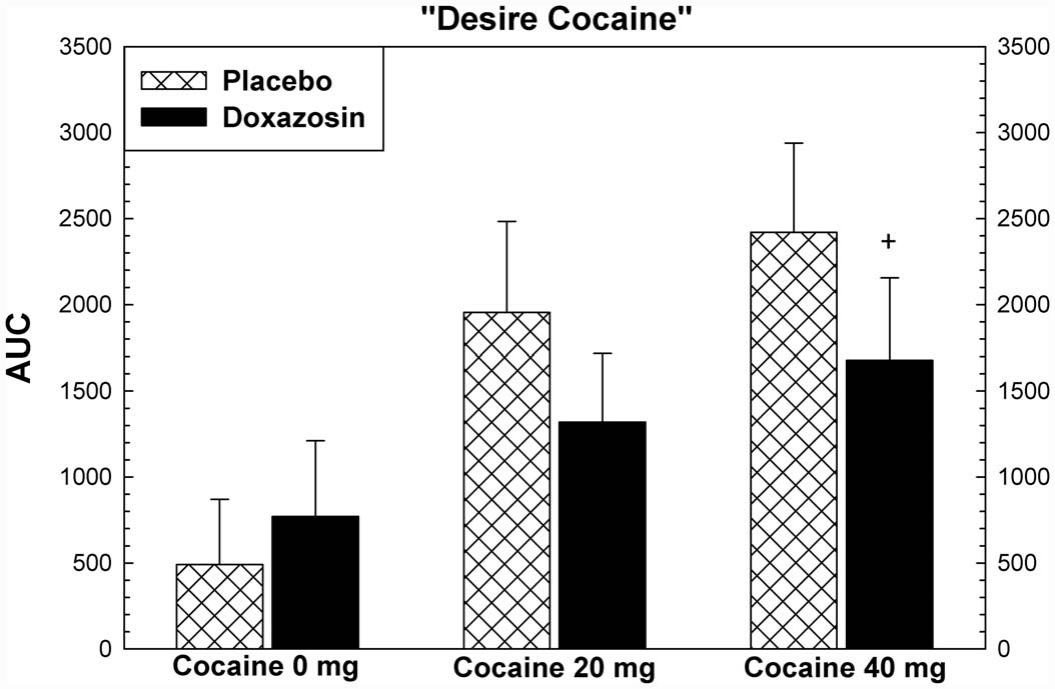
Subjective effects following cocaine dosing. Subjective effects ratings were collected using Likert scales ranging from 0 to 100, with 0 anchored as “not effects” and 100 anchored as “most ever”. ANOVA showed that there were significant effects (p<.05) of medication on ratings of “Stimulated”, “Like Cocaine”, “Desire Cocaine”, and “Likely to Use Cocaine”. Post hoc analysis showed that the statistically significant effects were observed following 20 mg cocaine with trend-level effects following 40 mg cocaine. * p<.05, + p<.10.

**Figure 8:**
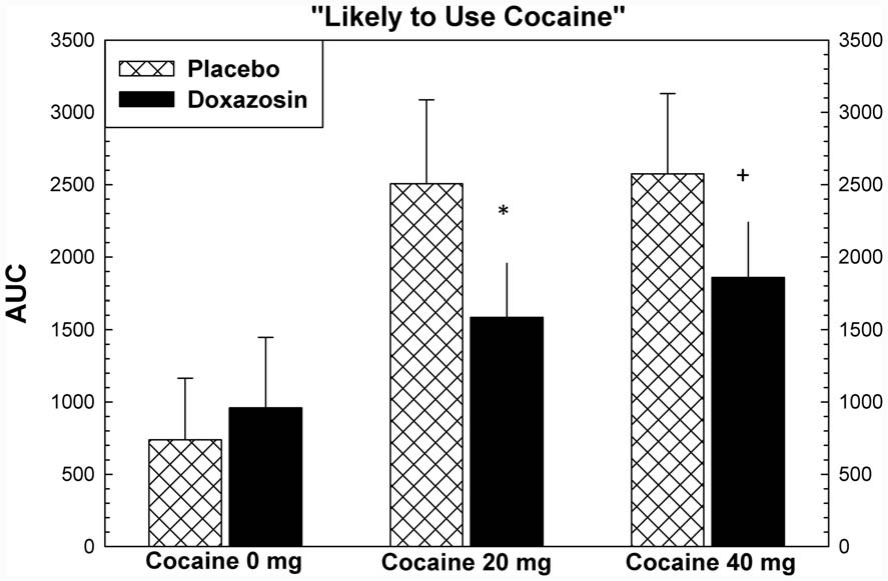
Subjective effects following cocaine dosing. Subjective effects ratings were collected using Likert scales ranging from 0 to 100, with 0 anchored as “not effects” and 100 anchored as “most ever”. ANOVA showed that there were significant effects (p<.05) of medication on ratings of “Stimulated”, “Like Cocaine”, “Desire Cocaine”, and “Likely to Use Cocaine”. Post hoc analysis showed that the statistically significant effects were observed following 20 mg cocaine with trend-level effects following 40 mg cocaine. * p<.05, + p<.10.

Cocaine produced increases in ratings for most subjective effects, including “high”, “any drug effect”, “stimulated”, and “good effects” (p<.001). There were significant effects of study drug treatment for ratings of “stimulated” (F = 7.41, p = .019), “like” (F = 5.17, p = .042), and “desire cocaine” (F = 5.47, p = .037), and “likely to use cocaine if had access” (5.57, p = .036). For each of these variables, the assumptions regarding normal distributions of data were met. For all but “like” the equal variance assumption was met as well. These are shown in figures 5, 6, 7, 8. *Post hoc* analysis (using the Holm-Sidak method) showed that doxazosin treatment reduced effects the effects of 20 mg cocaine on ratings of “stimulated” (t = 2.20, p = .035), “like” (t = 2.16, p = .037), and “likely to use cocaine if had access” (t = 2.29, p = .028). These analyses showed that doxazosin treatment produced trend-level reductions in the effects of 40 mg cocaine on ratings of “stimulated” (t = 1.78, p = .083), “desire cocaine” (t = 1.96, p = .059), and “likely to use cocaine if had access” (t = 1.78, p = .083). Doxazosin treatment did not have significant effects on ratings of “high”, “any drug effects” or other subjective effects ratings (not shown).

## Discussion

Doxazosin treatment was well tolerated, as was expected from earlier studies in other normotensive populations [21]. Doxazosin treatment had very modest effects on heart rate and blood pressure. The blood pressure effects of cocaine are likely mediated by sympathetic outflow and by effects of epinephrine and NE on peripheral vasculature and the observed effects of doxazosin on the cardiovascular effects of cocaine are consistent with this.

Doxazosin treatment significantly attenuated several of the positive subjective effects produced by cocaine, including ratings of “stimulated” and “like cocaine”, though the results for like should be interpreted with caution due to statistical limitations. Doxazosin also attenuated ratings of “likely to use” an index of craving. The magnitude of the effect was substantial for some of the variables (for example, the effect size d for reductions in “stimulated” during doxazosin treatment following administration of 20 mg cocaine was 0.84, which is considered large [22]).

The usual dose of doxazosin for the treatment of hypertension is 8–16 mg/d, which is several-fold higher than the dose we tested in this study, 4 mg/d. Further, though doxazosin 4 mg substantially attenuated many of the effects produced by 20 mg cocaine, this dose of doxazosin reduced the effects produced by 40 mg cocaine to a more modest extent. Higher doses of doxazosin would be expected to have a greater impact on the effects produced by a wider range of cocaine doses, including perhaps doses abused by cocaine users. These doses are thought to be in the 50 to 100 mg range, though there are no good data to base this estimate on. Nevertheless, that doxazosin antagonism of cocaine's was surmounted by the higher cocaine dose suggests a pharmacological dose-effect function and likely higher doses of doxazosin are needed for more complete antagonism.

These data are very much in keeping with data reported in rats by Zhang and colleagues [12], who found that prazosin pretreatment dose-dependently attenuated cocaine-induced reinstatement of extinguished cocaine-seeking behavior. The “reinstatement model” is frequently put forward as a model for craving induced by drug, stress, or other factors [23].

The report by Zhang and colleagues is somewhat at odds with older data reported by Woolverton [24] who found that prazosin did not alter responding maintained by cocaine. Prazosin treatment thus produced differential effects on cocaine reinstatement compared to reinforcing effects of cocaine. Consistent with this dissociation, we found that doxazosin treatment reduced indices reflecting desirability of cocaine, such as “like” and “likely to use cocaine with access”, without affecting indices reflecting euphoria, such as “high”. Euphoric effects are thought to relate to reinforcing effects [25], [26], though they need not necessarily do so [27].

The dampening effects of doxazosin on ratings of “stimulated” that we observed may reflect doxazosin's specific effects on noradrenergic neurotransmission. Cocaine inhibits the reuptake of NE with nearly the same potency that it inhibits the reuptake of DA [28]. The present data complement earlier preclinical research [7], and underscore the importance of noradrenergic mechanisms in mediating many of cocaine's effects.

It is not known precisely how doxazosin treatment modulates the subjective effects of cocaine. Noradrenergic α_1_Rs are expressed widely throughout the brain, most notably in the striatum and the prefrontal cortex (PFC) [29]. Acting within the PFC, doxazosin could block noradrenergically mediated release of DA in the fronto-accumbens circuit, blunting accumbal activation [30].

These data demonstrate, for the first time in humans, that an α_1_R receptor antagonist can attenuate several of the effects of cocaine. These findings parallel closely those previously reported in preclinical research, increasing confidence in the findings. Nevertheless, the sample size was relatively small and replication is needed. The dose of doxazosin used was at the low end of the therapeutic window, and higher doses should be tested. Other medications affecting adrenergic activation are used successfully in the treatment of hypertension [31] (also a noradrenergically-mediated phenomenon) and these might be assessed as possible treatments for cocaine dependence as well. Examples include other preparations of doxazosin, such as extended release doxazosin XL, α_2_R receptor agonists such as lofexidine, and other classes of antihypertensives, such angiotensin converting enzyme inhibitors (e.g. perindopril).

## Supporting Information

Protocol S1 **Trial Protocol.**
(DOC)

Checklist S1 **CONSORT Checklist.**
(DOC)
