# Supplementary material for: Noradrenergic α1 Receptor Antagonist Treatment Attenuates Positive Subjective Effects of Cocaine in Humans: A Randomized Trial
Source: PLoS One. 2012 Feb 3;7(2):e30854. doi: 10.1371/journal.pone.0030854 (PMC3272014; doi:10.1371/journal.pone.0030854)
Supplement: Protocol S1 — Trial Protocol. (DOC) [file pone.0030854.s001.doc]

[
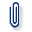
](javascript:openStandAloneWindow('../Shared/ViewAttachments.asp?protocol=235092'))[
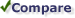
](https://brain.bcm.edu/esp1/reports/Human/ProtocolCompare.asp?protocol=235092)

|  |
| --- |
| Institutional Review Board for Baylor College of Medicine and Affiliated Hospitals |
|  |
| | **Protocol Number:**    **H-25442** | | --- | | Status:                        Approved | | Initial Submit Date:  7/15/2009 | | Approval Period:       6/21/2011 - 6/20/2012 | |
|  |
| **Section Aa:  Title & PI** |
| A1.  Protocol Title |
| |  | | --- | | |  | THE EFFECTS OF DOXAZOSIN ON THE CARDIOVASCULAR AND SUBJECTIVE EFFECTS OF COCAINE. VERSION 4.2 (JULY 2011) | | --- | --- | | |
| | **A2.  Principal Investigator** | | --- | |  | | |  | Name: | THOMAS F. NEWTON | Phone: | 713-791-1414 | | --- | --- | --- | --- | --- | |  | Id: | 161846 | Fax: | 713-794-7240 | |  | Department: | PSYCHIATRY & BEHAVIORAL SCIENCES | Email: | tnewton@bcm.tmc.edu | |  | Center: |  | Mail Stn: | BCM350 | | |
| | **A3.  Administrative Contact** | | --- | |  | | |  | Name: | NICOLETTE L VAN SLUIS | Phone: | 310-913-9308 | | --- | --- | --- | --- | --- | |  | Id: | 162727 | Fax: | 310-593-4193 | |  |  |  | Email: | sluis@bcm.tmc.edu | |  |  |  | Mail Stn: | BCM350 | | |
| A3a.  Financial Conflict of Interest |
| | Does the investigator have a financial interest in any non-Baylor sponsor or funding source for this research? | | --- | | |  | No | | --- | --- | | |
| A3b.  Cooperative Agreement |
| | Is this a cooperative agreement protocol? | | --- | | |  | No | | --- | --- | | |
| | Which institution is the IRB of record? | | --- | | |  | BCM: Baylor College of Medicine | | --- | --- | | |
|  |
| Section Ab:  General Information |
| | **A4.  Co-Investigators** | | --- | |  | | |  | Name: | RICHARD DE LA GARZA | Phone: | 713-791-1414 | | --- | --- | --- | --- | --- | |  | Id: | 162193 | Fax: | 713-794-7833 | |  | Department: | PSYCHIATRY & BEHAVIORAL SCIENCES | Email: | rg12@bcm.tmc.edu | |  | Center: |  | Mail Stn: | BCM350 | | |  | | |  | Name: | DAVID A NIELSEN | Phone: | 713-791-1414 | | --- | --- | --- | --- | --- | |  | Id: | 164409 | Fax: | 713-794-7240 | |  | Department: | PSYCHIATRY & BEHAVIORAL SCIENCES | Email: | nielsen@bcm.tmc.edu | |  | Center: |  | Mail Stn: | BCM350 | | |
| A5.  Funding Source: |
| |  | | --- | | |  | Organization:  NATIONAL INSTITUTE ON DRUG ABUSE (NIDA) | | --- | --- | | |
| A6a.  Institutions where work will be performed: |
| |  | | --- | | |  | Michael E. DeBakey Veterans Affairs Medical Center | | --- | --- | | |
| A6b.  Research will be conducted outside of the United States: |
| |  | | --- | | |  | Country:   Facility/Institution:   Contact/Investigator:   Phone Number:    If documentation of assurances has not been sent to the Office of Research, please explain: | | --- | --- | | |
| A7.  Research Category: |
| |  | | --- | | |  |  | | --- | --- | | |
|  |
| **Section B:  Exempt Request** |
| B.  Exempt From IRB Review |
| |  | | --- | | |  | Not Applicable | | --- | --- | | |
|  |
| Section C:  Background |
| |  | | --- | | |  | Human Laboratory Trial of Doxazosin for Cocaine Dependence: Accumulating evidence implicates noradrenergic (NE) systems in mediating the effects of stimulants (1-8). Mice lacking NE £\ -1 receptor mice show reduced sensitivity to cocaine and amphetamine (8, 9). Local depletion of prefrontal cortex (PFC) NE reduced rewarding effects of amphetamine and reduced amphetamine-induced dopamine (DA) release in the PFC and accumbens (7), suggesting that PFC NE contributes to the rewarding effects of stimulants. Treatment with the NE ƒÑ-1 antagonist prazosin has been shown to antagonize a variety of effects produced by cocaine and amphetamine. In rats, prazosin (1-2 mg/kg) significantly attenuated the locomotor activating effects produced by cocaine (10-12) and for amphetamine (4, 13, 14). Similar findings have been observed for the discriminative stimulus effects produced by cocaine and amphetamine in mice (15) and for food-maintained responding in pigeons (16). More recently, prazosin (0.3 mg/kg) reduced reinstatement of extinguished cocaine-seeking behavior in rats without affecting responding for food, suggesting that prazosin my blunt the motivational effects of drug cues (5). Clinical Experience with Alpha-1 Adrenergic Antagonists: Prazosin is a prototypical antagonist at NE £\-1 receptors. When first released the medication was thought to produce relaxation of smooth muscles in the vasculature. Since then however it has been determined that prazosin antagonizes norepinephrine and this mediates the antihypertensive effect (17). The medication has been assessed extensively for the treatment of hypertension (18). The initial dose is 1mg two or three times per day. The usual dose range is 6mg/day to 15mg/day, with some patients requiring up to 40mg/day in divided doses. The most common side effects are: dizziness 10.3%, headache 7.8%, drowsiness 7.6%, lack of energy 6.9%, weakness 6.5%, palpitations 5.3%, and nausea 4.9%. In most instances side effects have disappeared with continued therapy or have been tolerated with no decrease in dose of drug. More recently, prazosin has been successfully used to treat post traumatic stress disorder (19, 20). For this indication, prazosin was initiated at 1mg at bedtime and increased as needed to control symptoms to up to 15mg at bedtime by day 28. This approach resulted in no change in systolic and diastolic blood pressure compared to pretreatment measurements. Several subjects reported transient dizziness upon standing (9/14 in the prazosin group and 6/15 in the placebo group), but none reported syncope. Prazosin has a relatively short elimination half-life of 3.5 h (21). Longer-lasting cogeners (e.g. terazosin and doxazosin) are available, but there is no experience using these medications as treatments for cocaine dependence. The purpose of this study is to evaluate the effects of doxazosin on the cardiovascular and subjective effects of cocaine in a human laboratory study. Doxazosin was selected because it has a longer elimination half-life (22h) compared to terazosin (12h). The side-effect profile of doxazosin closely resembles that of prazosin. | | --- | --- | | |
|  |
| Section D:  Purpose and Objectives |
| |  | | --- | | |  | The purpose of the study is to asses the potential interactions between intravenous cocaine and doxazosin in cocaine dependent volunteers who are not seeking treatment. The study will evaluate the effects of doxazosin on the cardiovascular and subjective effects of cocaine in a human laboratory study.  Primary Objective: The primary objective is to determine the safety of treatment with doxazosin in cocaine-dependent volunteers by examining hemodynamic and subjective effects of administration of ascending doses of cocaine (0, 20mg, and 40mg) and a placebo dose during treatment with doxazosin. Secondary Objectives: • To evaluate the effect of doxazosin on the pharmacokinetics of intravenously administered cocaine • To determine effects of treatment with doxazosin, as compared to placebo, on subjective effects produced by administration of cocaine or placebo | | --- | --- | | |
|  |
| **Section E:  Protocol Risks/Subjects** |
| E1.  Risk Category |
| |  | | --- | | |  | (45 CFR 46.406) Category 3: Research involving greater than minimal risk and no prospect of direct benefit to the individual subject, but likely to yield generalizable knowledge about the subject's disorder or condition. | | --- | --- | | |
| E2.  Subjects |
| | Gender: | | --- | | |  | Both | | --- | --- | | |
| | Age: | | --- | | |  | Adult (18-64 yrs) | | --- | --- | | |
| | Ethnicity: | | --- | | |  |  | | --- | --- | | |
| | Primary Language: | | --- | | |  | English | | --- | --- | | |
| | Groups to be recruited will include: | | --- | | |  | Patients | | --- | --- | | |
| | Vulnerable populations to be recruited as subjects: | | --- | | |  |  | | --- | --- | | |
| | Vulnerable populations require special protections.  How will you obtain informed consent, protect subject confidentiality, and prevent undue coercion? | | --- | | |  |  | | --- | --- | | |
| E3.  Pregnant woman/fetus |
| | Will pregnant women be enrolled in the research? | | --- | | |  | No | | --- | --- | | |
| E4.  Neonates |
| | Will neonates be enrolled in the research? | | --- | | |  | No | | --- | --- | | |
| E5.  Children |
| | Will children be enrolled in the research? | | --- | | |  | No | | --- | --- | | |
|  |
| **Section F:  Design/Procedure** |
| F1.  Design |
| | Select one category that most adequately describes your research: | | --- | | |  | s) Drug, Phase I, Single Center | | --- | --- | | |
| | Discuss the research design including but not limited to such issues as: probability of group assignment, potential for subject to be randomized to placebo group, use of control subjects, etc. | | --- | | |  | Twenty participants will complete procedures during treatment with placebo and study medication, in random order, using a within-subjects, double-blind, placebo-controlled design. There will be a two week period between each phase. The dose of doxazosin needed to alter the effects of cocaine is unknown and preclinical animal studies have not been conducted. Because of this, initially we will study the effects of a low dose of doxazosin (4mg daily) compared to placebo daily. Because this class of medication needs to be titrated upward due to the potential for hypotension, treatment will begin at 1mg and increased by 1mg increments every three days until 4mg is reached. Placebo will be dosed identically. Blood pressure (resting and orthostatic) will be assessed daily prior to dosing. Cocaine (0, 20, and 40mg) will be administered during treatment with 4mg doxazosin/placebo. On day 10, cocaine will be administered in ascending dose (20mg followed by 40mg) with the 0mg dose randomly interspersed to maintain the blind. Cardiovascular measures and subjective effects ratings will be assessed following cocaine administration. Participants will be monitored for stability on days 11 and 12 and discharged from the hospital on day 13. Discharge will only occur for subjects who have a supine BP of 100/65 mm Hg or higher, a seated blood pressure of 90/60 mm Hg or higher, and an orthostatic change upon standing of < 20 mm Hg systolic or < 10 mm Hg diastolic.  If 4mg doxazosin treatment is well tolerated but is not associated with alterations in the effects of cocaine, a second group of subjects will be studied using the same sample size. Treatment will begin at 1mg and increased by 1mg increments every three days until 8mg is reached. All study procedures will be identical as described above. Placebo will be dosed identically. | | --- | --- | | |
| | Inclusion Criteria: | | --- | | |  | 1. Be English-speaking volunteers who are not seeking treatment at the time of the study; 2. Be between 18-55 years of age; 3. Meet DSM-IV TR criteria for cocaine dependence; participants may or may not meet criteria for nicotine dependence. Nicotine dependence is allowed but not required because most cocaine users smoke cigarettes. 4. Have a self-reported history of using cocaine by the smoked or IV route; 5. Have vital signs as follows: supine blood pressure > 100/65 mm Hg, a seated blood pressure of > 90/60 mm Hg and < 150/90 mm Hg, and an orthostatic change < 20 mm Hg systolic or <10 mm Hg diastolic on standing. Resting pulse must be < 90 bpm. 6. Have hematology and chemistry laboratory tests that are within normal (+/- 10%) limits with the following exceptions: a) liver function tests (total bilirubin, ALT, AST, and alkaline phosphatase) < 3 x the upper limit of normal, and b) kidney function tests (creatinine and BUN) within normal limits; 7. Have a baseline EKG that demonstrates clinically normal sinus rhythm, clinically normal conduction, and no clinically significant arrhythmias; 8. Have a medical history and brief physical examination demonstrating no clinically significant contraindications for study participation, in the judgment of the admitting physician and the principal investigator. | | --- | --- | | |
| | Exclusion Criteria: | | --- | | |  | 1. Meet DSM IV TR criteria for dependence on drugs other than cocaine or nicotine. 2. Have any history or evidence suggestive of seizure disorder or brain injury; 3. Have any previous medically adverse reaction to cocaine, including loss of consciousness, chest pain, or epileptic seizure; 4. Have neurological or psychiatric disorders, such as: • psychosis, bipolar illness or major depression as assessed by MINI; • organic brain disease or dementia assessed by clinical interview; • history of any psychiatric disorder which would require ongoing treatment or which would make study compliance difficult; • history of suicide attempts within the past year and/or current suicidal ideation/plan; 5. Have evidence of clinically significant heart disease or hypertension, as determined by the PI; 6. Have evidence of untreated or unstable medical illness including: neuroendocrine, autoimmune, renal, hepatic, or active infectious disease; 7. Have symptomatic HIV or are taking antiretroviral medication; 8. Be pregnant or nursing. Other females must either be unable to conceive (i.e., surgically sterilized, sterile, or post-menopausal) or be using a reliable form of contraception (e.g., abstinence, birth control pills, intrauterine device, condoms, or spermicide). All females must provide negative pregnancy urine tests before study entry, upon hospital admission, and at the end of study participation; 9. Have asthma or currently use theophylline or other sympathomimetics; 10. Have any other illness, condition, or use of psychotropic medications, which in the opinion of the PI and/or the admitting physician would preclude safe and/or successful completion of the study.  Criteria for Discontinuation Following Initiation Participants will be discharged if they have a positive breath test indicating use of alcohol or a urine test indicating illicit use of drugs while in the MED-VAMC, if they do not comply with study procedures, or if they do not tolerate the study drugs. Stopping criteria are detailed below.  Stopping Criteria Cocaine administration will not be initiated if there are clinically significant arrhythmias or if vital signs are outside of acceptable ranges, which are resting pulse < 130 bpm and blood pressure below 165mm Hg systolic and 100mm Hg diastolic. In addition, repeated doses of cocaine will not be administered (and the study physician will halt continued cocaine delivery) if there are behavioral manifestations of cocaine toxicity (agitation, psychosis, inability to cooperate with study procedures).   Stopping Criteria for Further Participation Subject participation will be terminated if any of the following events occur: 1. Systolic BP greater than 180 mm Hg sustained for 5 minutes or more; 2. Diastolic BP greater than 120 mm Hg sustained for 5 minutes or more; 3. Heart rate greater than (220 – age x 0.85) bpm sustained for 5 minutes or more. Note: if a single value exceeding the values above is detected, the readings will be repeated until it is determined whether or not the stopping criteria have been met.  Subject Selection Criteria Rationale Route of administration. Participants are required to have used cocaine by the IV or smoked route to avoid exposing participants to drugs by routes of administration that produce more intensive interoceptive effects than usually used by the participants. Prior experience with smoked cocaine is allowed (rather than restricting the population to those with experience with IV cocaine) because smoked cocaine reaches brain sites of action as rapidly as does intravenously administered cocaine and smoked cocaine produces effects that are comparable to IV cocaine. Speed of administration (and rate of delivery to brain) of stimulant drugs likely impacts subjective and cardiovascular effects, so smoked and intravenously administered cocaine produce similar subjective effects (22-26). | | --- | --- | | |
| F2.  Procedure |
| |  | | --- | | |  | Screening Interested candidates will receive an explanation of the study purpose and requirements. If still interested, the candidates will be allowed to review, inquire about, and sign the study informed consent form. After providing informed consent, subjects proceed to the screening assessment phase of the study. A urine drug toxicology screen will be conducted for drugs of abuse. Some of this sample will be shared with Rice university to develop new tests for drug detection. A saliva sample will also be collected for this purpose. Candidates deemed eligible based on initial screening assessments will be administered a MINI by a trained mental health professional, to determine if there are any psychiatric conditions that might exclude the potential subject from participation. Blood will be collected for analysis of hematology and chemistry tests. These assessments must be completed within 14 days before intake. At the initial screening visit, restrictions on use of alcohol, drugs of abuse, and medications will be reviewed. All subjects will be required to sign a written statement that they will abstain from use of cocaine or other psychostimulants throughout the duration of their participation in the trial.  All applicants for study participation will receive counseling as part of their participation, and will be advised that treatment for drug abuse is indicated and available. Applicants not participating in the study will receive treatment referral information as appropriate. At completion of their participation, study subjects will again be advised that treatment is indicated and available, and will be given treatment referral information and assistance.  Participants will reside on the Research Commons for the duration of the study (about 28 days total). Participants must refrain from illicit and prescription drug use, and we will confirm compliance by urine testing and breath alcohol level testing. Passes and monitoring. Passes will not usually be allowed, and visitors will not be permitted. Participants’ belongings will be searched upon arrival.  The study schedule outlined below may change by a day or two due to scheduling issues and/or safety reasons. We will ask participants to complete the protocol twice (once being randomized to placebo and once being randomized to doxazosin, but this wont be required.   General Procedures: Participants will be studied while residing on the Research Commons. Cocaine administration sessions will be conducted at approximately the same time of day for a given participant. Experimental sessions will be several hours in duration and will be conducted in a quiet room with research and medical staff present. The room will contain an experimental station, equipped with a response console attached to a personal computer and video monitor. Heart rate, EKG, and blood pressure will be recorded throughout experimental sessions using an automatic monitoring system.   Smoking, caffeine, and food. Caffeine and food will be prohibited during and 90 min before study sessions excepting a 1 hour break for lunch.   Day 1-10: The study participants will be randomized to receive either doxazosin or placebo. Doxazosin treatment will begin at 1mg and will be increased by 1mg increments every three days until 4mg is reached. Placebo will be dosed identically. Prior to dosing doxazosin, and at 1 hour intervals after each dose from hours 2 through 6 after dosing, sitting and supine orthostatic blood pressure will be assessed.  Cardiovascular monitoring and discontinuation criteria For doxazosin to be administered, the following criteria must be met: 1. supine blood pressure above 100/65 mm Hg, 2. a seated blood pressure of above 90/60 mm Hg, 3. and an orthostatic change smaller than 20 mm Hg systolic or smaller than 10 mm Hg diastolic on standing. The dose will not be increased until the participant has met criteria for dosing and has received 3 doses at a given dose level.  a. Participants who do not meet the criteria above at any time during hours 2 through 6 after dosing will remain supine until the next BP assessment. b. Participants who do not meet the criteria above consistently from hours 2 through 6 after any dosing will not have their dose of doxazosin increased and will remain at the prior dosing level. c. Participants who do not meet the requirements for dosing increase (supine blood pressure > 100/65 mm Hg, a seated blood pressure of > 90/60 mm Hg, and an orthostatic change < 20 mm Hg systolic or <10 mm Hg diastolic on standing) for 6 days will have medication discontinued and they will be discontinued from the study.   Day 10: Cocaine 20mg, 40mg and matched placebo saline will be administered via IV push by the study physician on day 10, at hourly intervals, with the placebo dose randomly interspersed to maintain the blind. To ensure that subjects will not be at risk from cocaine, the resting pulse must be < 90 bpm and the blood pressure must be < 150 mmHg systolic and < 90 mmHg diastolic immediately prior to dosing. Heart rate and blood pressure measurements will be made prior to cocaine dosing and at 15-minute intervals thereafter until 30 minutes following the final dose. Visual-analogue scale ratings of “any cocaine effect”, “high”, “good effects”, “bad effects”, “like cocaine”, “desire cocaine”, “depressed”, “anxious”, “stimulated”, and “if you had access to cocaine right now, how likely would you be to use it?” will be collected at -15 min, 5 min, 10 min, 15 min, 20 min, 30 min, and 45 min after cocaine dosing. Participants will remain seated during experimental sessions except for brief visits to the lavatory.   All infusions will be administered in single-blind fashion. All experimental procedures will be conducted in a hospital room located in the GCRC. Infusions will be administered over 2 minutes by I.V. push. Vital signs must remain within values specified under Stopping Criteria for initiation of cocaine administration. An ACLS-certified physician will be present from 15 min before drug administration until at least 1 hour after cocaine administration. The physician will be available in house and on pager for at least 4 hours after drug administration. Heart rate will be measured using continuous ECG from 15 min prior to cocaine or placebo dosing until 2 hours after dosing. Blood pressure will be assessed at frequent intervals from 15 min prior to cocaine or placebo dosing until 2 hours after dosing.   Discharge Participants will be monitored for stability on days 11 and 12 and discharged from the hospital on day 13. Discharge will only occur for subjects who have a supine BP of 100/65 mm Hg or higher, a seated blood pressure of 90/60 mm Hg or higher, and an orthostatic change upon standing of < 20 mm Hg systolic or < 10 mm Hg diastolic. Prior to discharge subjects will have a physical examination and vital signs, and complete the Beck Depression Inventory-II. An assessment of adverse events will be completed.  Participants will have the choice of returning two weeks later to complete study procedures.   Stopping Rules Doxazosin Participants who do not meet the criteria listed above (supine blood pressure above 100/65 mm Hg, a seated blood pressure of greater than 90/60 mm Hg, and an orthostatic change smaller than 20 mm Hg systolic or smaller than 10 mm Hg diastolic on standing) at any time during hours 2 through 6 after dosing will remain supine until the next BP assessment. Participants who do not meet the criteria above consistently from hours 2 through 6 after any dosing will not have their dose of doxazosin increased and will remain at the prior dosing level. Participants showing syncope or dizziness that does not respond to sitting down will be discharged from the study.  Stopping Criteria for Further Participation Participants who do not meet the requirements for dosing increase for 6 days will have medication discontinued and they will be discontinued from the study.  Cocaine Cocaine will not be administered if there are clinically significant arrhythmias or if vital signs are outside of acceptable ranges, which are resting pulse below 90 bpm and blood pressure below 150mm Hg systolic and 90mm Hg diastolic. In addition, repeated doses of cocaine will not be administered (and the study physician will halt continued cocaine delivery) if there are behavioral manifestations of cocaine toxicity (agitation, psychosis, inability to cooperate with study procedures).   Stopping Criteria for Further Participation Subject participation will be terminated if any of the following events occur: 1. Systolic BP above 180 mm Hg sustained for 5 minutes or more; 2. Diastolic BP above 120 mm Hg sustained for 5 minutes or more; 3. Heart rate above (220 – age x 0.85) bpm sustained for 5 minutes or more. Note: if a single value exceeding the values above is detected, the readings will be repeated until it is determined whether or not the stopping criteria have been met.  Adverse event assessment and management. We will assess participants’ well-being daily. Spontaneously reported symptoms or complaints will be recorded and reported to the IRB and NIDA and the FDA if events are classified as serious. Specific guidelines have been developed to guide assessment and intervention in the event of cardiovascular toxicity, such as malignant hypertension, ventricular arrhythmia, chest pain, etc., and are in use for our other studies involving cocaine administration. These guidelines are intended to support rather than supersede clinical judgment. If needed, a code team responds. Participants needing to be transferred to a medical or ICU ward can be, though this has never been necessary.   Assessments see attached | | --- | --- | | |
|  |
| **Section G:  Sample Size/Data Analysis** |
| G1.  Sample Size |
| | How many subjects (or specimens, or charts) will be used in this study? | | --- | | |  | Local: 200              Worldwide: 200 | | --- | --- | | |
| | Please indicate why you chose the sample size proposed: | | --- | | |  | Rationale for sample size: The power analysis is based on a published report on the subjective and physiological effects of intravenous cocaine (Foltin & Fischman, 1991). One of the commonly used items “Feel High” was chosen for power analysis. We anticipate a reduction by one-half as a clinically significant treatment effect. For a sample size of 20, with an alpha level of 0.05, a paired t-test indicates power of approximately 0.80.   To have twenty completers, we may need to enroll up to 30 participants and thus screen/consent 200 potential subjects. | | --- | --- | | |
| G2.  Data Analysis |
| | Provide a description of your plan for data analysis. State the types of comparisons you plan (e.g. comparison of means, comparison of proportions, regressions, analysis of variance). Which is the PRIMARY comparison/analysis? How will the analyses proposed relate to the primary purposes of your study? | | --- | | |  | Descriptive statistics will be used to summarize basic demographic information and drug use variables. Placebo vs. medication groups will be compared using one–way ANOVA. If significant differences between treatment groups are identified, then these variables can be included in further analyses as covariates. General linear models for repeated measures will be used to assess placebo vs. medication effects on cardiovascular responses, subjective effects, or reinforcing effects. | | --- | --- | | |
|  |
| **Section H:  Potential Risks/Discomforts** |
| H1.  Potential Risks/Discomforts |
| | Describe and assess any potential risks/discomforts and assess the likelihood and seriousness of such risks: | | --- | | |  | Potential risks of the study are those conferred by administration of doxazosin, administration of cocaine, and administration of the combination of the two. Other risks include those associated with residence on the research commons such as inadvertent breach of confidentiality.   Risks of Study drugs: Risks of cocaine; The risks from cocaine are raised or lowered blood pressure and heart rate, tremors, vomiting, convulsions, stroke, myocardial infarction, and in very rare instances, possibly death. If taken in high doses, cocaine can cause paranoia and delusions, but if taken in the doses used in this study, these effects are uncommon. The most frequent effects are changes in blood pressure and heart rate; the other effects are quite rare.   Risks associated with doxazosin administration; Dizziness, headache, drowsiness, lack of energy, weakness, palpitations, and nausea. In most instances side effects have disappeared with continued therapy or have been tolerated with no decrease in dose of drug. Less frequent adverse reactions which are reported to occur in 1-4% of patients are: Gastrointestinal: vomiting, diarrhea, constipation. Cardiovascular: edema, orthostatic hypotension, dyspnea, syncope. Central Nervous System: vertigo, depression, nervousness. Dermatologic: rash. Genitourinary: urinary frequency. EENT: blurred vision, reddened sclera, epistaxis, dry mouth, nasal congestion.  Risks of placebo administration; There are no known serious health risks to treatment with placebo.  Risks of Blood Collection The risks of inserting a needle into a vein may involve (1) pain from insertion of the needle; (2) lightheadedness; (3) fainting; (4) hematoma (like a bruise) at the site of the needle insertion; (5) inflammation of the vein; (6) clotting of the vein; (7) rarely, infection where the needle enters the skin, or (8) rarely, an allergic reaction to the tape applied afterwards.  Risks of ECG recording ECG recordings are safe. Some people may develop mild irritation due to the adhesive used to hold the electrodes on. The gel may also require washing your hands and chest after the study. Rarely, there may be an allergic reaction to ECG electrode adhesive, which might result in redness or swelling.  Other Risks and Discomforts   Some people get depressed after they stop using cocaine. Subjects will be monitored closely for symptoms of depression. The study team consists of psychiatrists and physicians who are all experienced in the field of drug abuse and they can provide counseling and/or medications if necessary. If subjects demonstrate severe depression, appropriate medical action will be taken to keep the subject safe. He/she may need to leave the study and will be referred to appropriate care, if they wish. No subjects with severe depressive symptomatology will be allowed to leave the hospital until it is established that they are safe (i.e., no suicidal ideation is present).  During the entry visit patients will be asked questions about their medical history and lifestyle and drug use. Some of these questions may be personal, and patients may feel uncomfortable or embarrassed. The questions will be asked in a private room such that no one besides the study staff will know the answers.  There is the risk of unauthorized disclosure of confidential information and possible unwanted encounters with friends or acqaintances in the treatment setting.  Visits to the study center for study procedures may be time consuming and inconvenient. Participation in the study will last about 28 days.  There may be risks and discomforts associated with participation in this study which are currently unforeseeable  Minimizing Risks: Subjects will be given a complete physical exam and medical history at entry to determine any reason why the study procedures should not be administered. Subjects will be instructed in the consent form and verbally to immediately report any adverse experiences that may arise. Subjectswill be instructed verbally and in the consent form to report any changes in concurrent medications and to check with their primary care physicians for any reasons they should not enter or continue on the study. Study sites will forward any and all adverse reactions information to the sponsor for analysis.  Cocaine is a strong sympathomimetic and has been associated with adverse cardiovascular events, including myocardial infarction and stroke, when taken illicitly. While these consequences have never been observed following controlled experimental administration, we will limit these potential risks in several ways: we will enroll only physically healthy subjects with no evidence of cardiovascular disease; we will administer relatively small doses of cocaine (up to 40mg); and we will monitor subjects closely in a medical hospital throughout the duration of action of the drug; and we have an intervention plan in place to deal with any adverse events that may occur.  We have extensive experience safely administering individual doses of 40mg cocaine. These doses are modest compared to amounts that participants in these studies have reported using daily; typical daily dosing patterns are on average 250mg to 500mg or more. Based on evidence from other laboratories, the risks associated with repeated cocaine administration are not substantially greater than those produced by single doses (Ward et al 1997a; Ward et al 1997b). The mechanisms that limit cardiovascular responses following repeated dosing are not known, but probably involve the development of acute tolerance to the cardiovascular effects of cocaine. We will carefully monitor participants and will not administer additional doses of cocaine until cardiovascular parameters have returned to preset limits, further safeguarding subjects. In the unlikely event that a subject experiences a medical emergency, the research commons is prepared to respond to any situation that may arise. It is located 5th floor of MEDVAC near Dr. Newton's office. The general and psychiatric emergency rooms are located minutes away on the 1st floor of the hospital. There is a 24-hour medical emergency code team in the hospital. There is a 24-hour medical emergency code team in the hospital. A fully equipped crash cart is located on the unit.  It is our standard to obtain ECGs during screening, upon admission, and prior to discharge. Dr. Newton will monitor all ECGs. He has ACLS certification and has 14 years experience monitoring ECGs for studies involving cocaine administration.   Safety data will be reviewed regularly by a data and safety monitoring board. The board will be blinded to subjects' actual treatment assignments for the safety data. Reports from the DSMB will be sent to the site investigator for transmission to the appropriate IRB, in accordance with NIH policy.  Strict go/no-go criteria will be utilized for the infusions, and before and after each challenge session, subjects will be monitored using continuous heart rate, blood pressure, and EKG readings.  If suicidal ideation is identified in any subjects, the study physician will be contacted immediately and meet with the subject within one hour. If the participant expresses having a plan or has a history of previous suicide attempts, the subject will not be left alone until a study physician has met with him/her. If no physician is available 911 will be called to assess the situation. The studyphysician will determine the severity of the subject's suicidal intentions. If the study physician feels that the subject's risks are low then s/he will be given referrals to counselors, hotlines and clinics where s/he can seek further help. However, if the study physician determines that the subject's suicidal risks are high, he/she will develop a plan for safety with the subject, including but not limited to: contacting the participant's personal physician, encouraging the subjects to confide in family members or close friends,giving suicide hotline information, and providing appropriate referrals.  Sterile techniques will be utilized for blood draws and injections. Standard nursing techniques will be used to make the subjects as comfortable as possible. The entry visit interview will be administered in private with the utmost sensitivity to the subject. Prior to discharge an MD will evaluate the patients' medical (including vitals) and psychiatric status and determine if it is safe for them to be discharged. | | --- | --- | | |
| H2.  Data and safety monitoring plan |
| | Do the study activities impart greater than minimal risk to subjects? | | --- | | |  | Yes  NOTE:  The answer to the questions in H2 requires the completion of the form: 'Section H – Data and Safety Monitoring Plan' as an attachment in Section S. | | --- | --- | | |
| H3.  Coordination of information among sites for multi-site research |
| | Is the BCM Principal Investigator acting as the SPONSOR-INVESTIGATOR for this multi-site research? | | --- | | |  | No or Not Applicable | | --- | --- | | |
| | Is BCM the COORDINATING CENTER for this multi-site research? | | --- | | |  | No or Not Applicable | | --- | --- | | |
|  |
| Section I:  Potential Benefits |
| | Describe potential benefits to be gained by the individual subject as a result of participating in the planned work. | | --- | | |  | The benefits to the participant include free testing (medical exam, lab tests, EKG) and possible discovery of reversible disease, which could be reported to a health care provider with the subjects written request and consent. There is also the opportunity to participate in research that may increase subjects' knowledge and understanding of their drug dependence and its interaction with their health status. All drug-abusing applicants for study participation will receive counseling about drug dependence and advised that treatment for drug abuse is indicated and available. Applicants not participating in the study will receive treatment referral information as appropriate. At the completion of their participation, study participants will again be advised that treatment is indicated and available, and will be given treatment referral information and assistance. In our protocols subjects are in a controlled and drug-free environment for a reasonable amount of time that oftentimes encourages them to take the first step towards either cutting down cocaine use on their own, or getting treatment. | | --- | --- | | |
| | Describe potential benefits to society of the planned work. | | --- | | |  | Knowledge to be gained from this research may increase our understanding of the subjective and physiological effects of cocaine and teach us more about the cocaine using population. The information on the effect of doxazosin on cocaine use behavior, and any tendency towards treatment efficacy enhances prospects for medication development for treatment of drug abuse. Information concerning the effects of cocaine on health and potential treatments is increasingly important in view of the rising prevalence of stimulant use, especially among the young people in our society. | | --- | --- | | |
| | Do anticipated benefits outweigh potential risks? Discuss the risk-to-benefit ratio. | | --- | | |  | The primary risks of this study are those of possible adverse reactions to the study drugs, cocaine and disulfiram. We have extensive experience administering cocaine safely in the laboratory. The doses of cocaine used are modest, the safety screening and monitoring procedures are careful, and there have been no significant prior serious adverse events with these procedures.   Doxazosin is a marketed product with which there is extensive experience and little indication of significant risk.   There is the risk of a breach of confidentiality regarding study records, but this is unlikely, since staff is well trained and experienced in this area.  The study does not offer direct therapeutic benefit to participants. However, because it is directed toward the identification and development of effective treatment for cocaine abuse and dependence, it does offer the potential of future benefit to this same population group.  Overall, the risks are significant, but appropriate safety precautions have been taken. Since there is a potential societal health benefit, we believe the risk/benefit ratio is favorable. | | --- | --- | | |
|  |
| **Section J:  Consent Procedures** |
| J1.  Waiver of Consent |
| | Will this research require a waiver of consent and authorization? | | --- | | |  | No | | --- | --- | | |
| | Will additional pertinent information be provided to subjects after participation? | | --- | | |  | No | | --- | --- | | |
| | Explain why providing subjects additional pertinent information after participation is not appropriate. | | --- | | |  |  | | --- | --- | | |
| J1a.  Waiver of requirement for written documentation of Consent |
| | Is this research subject to FDA regulations? | | --- | | |  | No | | --- | --- | | |
| | Explain how the research involves no more than minimal risk to the participants, and the specifics demonstrating that the research does not involve procedures for which written consent is normally required outside of the research context. | | --- | | |  |  | | --- | --- | | |
| | Explain how the only record linking the participant and the research would be the consent document, and how the principal risk would be potential harm resulting from a breach of confidentiality, and how each participant will be asked whether he or she wants documentation linking the participant with the research and their wishes will govern. | | --- | | |  |  | | --- | --- | | |
| J2.  Consent Procedures |
| | Who will recruit subjects for this study? | | --- | | |  | PI Research subject (ex - recruitment of family member into genetic studies) PI's staff | | --- | --- | | |
| | Describe how research population will be identified, recruitment procedures, and consent procedures in detail. | | --- | | |  | Potential subjects will be self-identified as non-treatment seeking cocaine users and recruited using advertisements in certain newspapers, on radio stations, websites, and community locations (e.g., phone booths, needle exchanges), as well as through referrals from within as well as from outside organizations, and from past research participants. Past research participants are an important source of referrals for us and they frequently ask us for business cards or other items that they can give to their friends and acquaintances. Advertisements are designed to attract cocaine users interested in participation in research on substance abuse in exchange for monetary compensation.   Recruitment will also be accomplished through referrals and word-of-mouth. Recruitment of “hidden populations” such as users of illicit drugs is difficult with standard methods such as advertisements/flyers. Though we will continue to recruit via the methods already stated, we will also use a method of recruitment called Respondent-Driven Sampling (RDS; Booth et al., 2006; Borders et al., 2008; Draus et al., 2005; Heckathorn, 2002, Wang et al., 2004). This variation of snowball samplingallows participants to serve as “seeds” for future participants, and has been found to be an effective recruitment method for “hidden populations” such as illicit drug users. Each participant will have the opportunity to refer other individuals who may be appropriate for the study. A participant who refers another individual who enrolls and completes this single-session study will receive a referral fee ($20). This fee will be processed on a monthly basis and the referring individual will receive a check within two weeks of processing. We recognize that this may result in a non-random sample. However, given the challenges of recruitment from our target population, and the significant value of obtaining discounting measures from this population, we believe this potential tradeoff is justified and appropriate. Furthermore, RDS has been found to result in sample characteristics that are stable and accurately reflect population characteristics following successful recruitment waves (Heckathorn, 2002, Wang et al., 2004).  Screening will be done by a research assistant and/or clinician and take about 2 hours to obtain informed consent, cover explanation of the study, evaluation of exclusion criteria, screening for DSM-IV criteria for MA, cocaine or other drug dependence, and administration of the intake package (see assessments section). All subjects who volunteer to participate will be adults aged 18-55, and will be judged both mentally and emotionally competent and capable of providing informed consent. This judgment will be made by study staff who are knowledgeable about and experienced in working with the drug abusing population. If there is any doubt about the decisional capacity of a potential participant, study staff will consult with the principal investigator (Dr. Newton is a clinical psychiatrist) and/or the study physician.  At several times during the consent process, participants will be informed that other forms of treatment, including abstinence-based treatment, are available and they will be referred if they so desire. The study staff will strongly encourage the subjects to take the consent home with them, review it and the study with family and/or friends, and to ask the study staff any questions they may have. After consent has been obtained, a physical and laboratory examination will include ECG and blood evaluation (chemistries, hematology). A study physician will then interview the subject for psychiatric diagnosis and review of medical data, and verify that the research subject understands the research procedures and the risk/benefit before study entry. Subjects will not be allowed to provide consent in an intoxicated state. | | --- | --- | | |
| | Are foreign language consent forms required for this protocol? | | --- | | |  | No | | --- | --- | | |
| J3.  Privacy and Intrusiveness |
| | Will the research involve observation or intrusion in situations where the subjects would normally have an expectation of privacy? | | --- | | |  | No | | --- | --- | | |
| J4.  Children |
| | Will children be enrolled in the research? | | --- | | |  | No | | --- | --- | | |
| J5.  Neonates |
| | Will non-viable neonates or neonates of uncertain viability be involved in research? | | --- | | |  | No | | --- | --- | | |
| J6.  Consent Capacity - Adults who lack capacity |
| | Will Adult subjects who lack the capacity to give informed consent be enrolled in the research? | | --- | | |  | No | | --- | --- | | |
| J7.  Prisoners |
| | Will Prisoners be enrolled in the research? | | --- | | |  | No | | --- | --- | | |
|  |
| Section K:  Confidentiality |
| | Will research data include health information by which subjects can be identified? | | --- | | |  | Yes | | --- | --- | | |
| | Where will research data be kept? How will such data be secured? | | --- | | |  | This study will acquire, use and create individually identifiable health information (known as Protected Health Information or PHI). Because of the federal law known as HIPAA (Health Insurance Portability and Accountability Act), which went into effect on April 14, 2003 and is aimed at protecting an individual’s health information, all individuals who are eligible and agree to participate in this research study will be required to sign a HIPAA research authorization prior to participation. If an individual refuses to sign the HIPAA research authorization, they cannot participate in this study.  Confidentiality will be protected for both physical and digital records. All research information will be stored in either locked files or secure computers, which will also be kept in locked rooms at all times. Only authorized research staff will have access to the information gathered in this study.  Confidentiality Information Patient research records will be kept as confidential as possible. All research information will be kept in either locked files or secure computers, which will also be kept in locked rooms, at all times. Patient identity will not be revealed in any reports or publications resulting from this study. | | --- | --- | | |
| | Who, besides the PI, the study staff, the IRB and the sponsor, will have access to identifiable research data? | | --- | | |  | Only authorized research staff will have access to the information gathered in this study | | --- | --- | | |
| | Will you obtain a Certificate of Confidentiality for this study? | | --- | | |  | Yes | | --- | --- | | |
| | Please further discuss any potential confidentiality issues related to this study. | | --- | | |  | We will be collecting sensitive information on participants. We will be collecting data on drug use habits, legal status, and psychiatric history. All data and information collected from subjects will be kept in a secure location and only study staff and the investigator will have access to it. | | --- | --- | | |
|  |
| Section L:  Cost/Payment |
| | Delineate clinical procedures from research procedures. Will subject's insurance (or subject) be responsible for research related costs? If so state for which items subject's insurance (or subject) will be responsible (surgery, device, drugs, etc). If appropriate, discuss the availability of financial counseling. | | --- | | |  | The subject/subject's insurance will not be responsible for any study-related costs. | | --- | --- | | |
| | If subjects will be paid (money, gift certificates, coupons, etc.) to participate in this research project, please note the total dollar amount (or dollar value amount) and distribution plan (one payment, pro-rated payment, paid upon completion, etc) of the payment. | | --- | |
| | Dollar Amount: | | --- | | |  | 1540 | | --- | --- | | |
| | Distribution Plan: | | --- | | |  | Participants will be paid $40 in gift certificates for the completion of the screening. The payment schedule is shown below. $50 is the average compensation provided for each day, however, compensation increases over the course of the study to make up for the increasing difficulty participants may have abstaining from illicit cocaine use. If a participants leaves the study early, payment will be prorated.   Day 0 - $20 Day 1 - $20 Day 2 - $25 Day 3 - $30 Day 4 - $35 Day 5 - $40 Day 6 - $45 Day 7 - $50 Day 8 - $55 Day 9 - $60 Day 10 - $80 Day 11 - $100 Day 12 - $50 Day 13 - $50 total $660  In addition, participants will receive an extra $40 on Monday, which can be any study day, depending on the day of entry.This is to compensate for staying over the weekend. Payment on Day 11 is larger to serve as a study completion bonus. Subjects completing both phases will receive $1400 plus a $100 bonus. | | --- | --- | | |
|  |
| Section M:  Genetics |
| | How would you classify your genetic study? | | --- | | |  | DNA diagnostic study | | --- | --- | | |
| | Discuss the potential for psychological, social, and/or physical harm subsequent to participation in this research. Please discuss, considering the following areas: risks to privacy, confidentiality, insurability, employability, immigration status, paternity status, educational opportunities, or social stigma. | | --- | | |  | The use of patient blood for genetic testing raises special issues of confidentiality, because it is conceivable that information about patient genes could be used against patient if the wrong people knew this information. For example, an insurance company could try to deny benefits, or an employer could try to deny employment, if it became known that patient carried certain genes. To reduce this possibility, the following specific measures will be taken to protect patient confidentiality:  1. The genetic testing of patient DNA is for research purposes only. No results of genetic testing from this study will appear in the patient medical record.  2. Genetic test results will not be made available to patients or their physicians.   3. Data will be stored in a collective database kept by David Nielsen, PhD in the Division of Addiction Psychiatry, Department of Psychiatry and Behavioral Sciences. We will store de-identified phenotypic and genotypic data in a state-of-the-art database system maintained by Baylor College of Medicine Information Technology Department. This system, called the PsychGen-DB, is a robust informatics system that integrates clinical, genetic, demographic, and security data to aid our research into the biology and genetics of psychiatric diseases. The PsychGen-DB system is a two-tiered system consisting of an Oracle back-end for data management and storage, and a web-based graphical user interface front-end application for data entry, browsing, and retrieval. The database will be accessible through an application front end within the BCM internal network. No access to the database will be granted from outside the BCM network. The application front-end uses BCMauth_appl. BCMauth_appl uses LDAP to determine a user’s access status on the BCM network. Additionally, BCMauth_appl limits users’ access based on a role assigned by the PsychGen-DB administrator. Additionally, access into PsyGen-DB database is controlled through the use of database privileges and user roles. The database administrator is responsible to administer the PsyGen-DB and will provide explicit rights only to authorized users with an authorized BCM account. The PsychGen-DB system is built on top of Oracle 10gR2. The PsychGen-DB system runs in an isolated instance of Oracle on an IBM 9117-MMA Server (64 Bit, 6 Processors Power 6 4.2GHZ, 32 GB physical memory, and AIX 5.3 operating system). This is a scalable system giving us the flexibility to grow as dictated by our needs. Contingency of our systems operations is achieved by using Hitachi Redundant Array of Independent Disk (RAID) storage. The PsychGen-DB system is backed up daily to disk using Oracle’s Recovery Manager and to a tape library system using Legato Networker Module. System administration and security patch maintenance occur once a month and is performed by a System Administrator from Baylor College of Medicine Information Technology Department. The database and application server(s) are housed in the BCM data center located at One Baylor Plaza, Houston, TX. The server(s) are protected by an Uninterruptible Power Source (UPS) and backup gas generator.   While all efforts are aimed at protecting patient blood and/or DNA samples, there remains the possibility that VA could be compelled by a court or a law enforcement agency to produce such samples. We have been collecting DNA samples for years and no outside agency has ever tried to gain access to any research participant's blood or DNA samples. We believe that the risk of this happening to a patient sample is extremely small. | | --- | --- | | |
| | Will subjects be offered any type of genetic education or counseling, and if so, who will provide the education or counseling and under what conditions will it be provided? If there is the possibility that a family's pedigree will be presented or published, please describe how you will protect family member's confidentiality? | | --- | | |  | N/A | | --- | --- | | |
|  |
| **Section N:  Sample Collection** |
| **SAMPLE:   Other: saliva and/or blood** |
| | What is the purpose of the sample collection? | | --- | | |  | Saliva/blood will be collected and DNA will be extracted (Oragene, DNA Genotek, Ottawa CANADA) and genotyped for key targets predicted to be important in the rewarding effects produced by stimulant drugs (e.g., DAT, DAD2, COMT, DBH, etc). | | --- | --- | | |
| | For blood draws, specify the amount drawn, in teaspoons, at each visit and across the course of the subjects entire participation time. | | --- | | |  | 5cc | | --- | --- | | |
| | Is there the possibility that cell lines will be developed with this sample?No | | --- | |
| | Sample will be obtained from: | | --- | | |  | Other: participant | | --- | --- | | |
| | Will the sample be stripped of identifiers? | | --- | | |  | No | | --- | --- | | |
| | **If sample will be released outside the hospital:** | | --- | |
| | Will sample be released to anyone not listed as an investigator on the protocol? Will the information be identifiable, coded or de-identified? | | --- | | |  | no | | --- | --- | | |
| | Will sample material be sold or transferred to any third parties? Will the information be de-identified? | | --- | | |  | no | | --- | --- | | |
| | **If sample will be banked for future use:** | | --- | |
| | Where will the sample be banked and for how long? | | --- | | |  | no | | --- | --- | | |
| | Does the banking institution have an approved policy for the distribution of samples? | | --- | | |  | n/a | | --- | --- | | |
| | **If the entire sample will NOT be used during the course of this research study:** | | --- | |
| | Will the remaining tissue be discarded? If not what will be done with the remaining sample after study completion and how long will the sample be kept? | | --- | | |  | DNA will be stored for future analysis. Genetic data will be used to characterize the COC-dependent population. | | --- | --- | | |
| | Will samples be made available to the research subject (or his/her medical doctor) for other testing? | | --- | | |  | No | | --- | --- | | |
| | **If a subject withdraws from the study:** | | --- | |
| | Will subject have the option to get the remaining portion of their sample back? | | --- | | |  | No | | --- | --- | | |
| | Will samples be destroyed? If not, will they be kept anonymously? What will happen to the sample if the subject revokes authorization? | | --- | | |  | Data obtained will be destroyed if subject revokes authorization. | | --- | --- | | |
| | Will data obtained from their sample be deleted? What will happen to the sample if the subject revokes authorization? | | --- | | |  | Data obtained will be destroyed if subject revokes authorization. | | --- | --- | | |
| | Will study data or test results be recorded in the subject's medical records? | | --- | | |  | No | | --- | --- | | |
| | Will results of specific tests and/or results of the overall study be revealed to the research subject and or his/her doctor? | | --- | | |  | no | | --- | --- | | |
| | Please identify all third parties, including the subject's physician, to receive the test results. | | --- | | |  | Results of specific tests and/or results of the overall study will NOT be revealed to the research subject or his/her doctor. | | --- | --- | | |
|  |
| **SAMPLE:  Blood** |
| | What is the purpose of the sample collection? | | --- | | |  | During screening, blood will be collected in a serum separation evacuated venous blood collection tubes (e.g., VacutainerTM) and serum separated according to standard procedures. Standard hematology and chemistry tests will be performed. | | --- | --- | | |
| | For blood draws, specify the amount drawn, in teaspoons, at each visit and across the course of the subjects entire participation time. | | --- | | |  | 2 tbls | | --- | --- | | |
| | Is there the possibility that cell lines will be developed with this sample?No | | --- | |
| | Sample will be obtained from: | | --- | | |  | Other: participant | | --- | --- | | |
| | Will the sample be stripped of identifiers? | | --- | | |  | No | | --- | --- | | |
| | **If sample will be released outside the hospital:** | | --- | |
| | Will sample be released to anyone not listed as an investigator on the protocol? Will the information be identifiable, coded or de-identified? | | --- | | |  | No | | --- | --- | | |
| | Will sample material be sold or transferred to any third parties? Will the information be de-identified? | | --- | | |  | No | | --- | --- | | |
| | **If sample will be banked for future use:** | | --- | |
| | Where will the sample be banked and for how long? | | --- | | |  | NO | | --- | --- | | |
| | Does the banking institution have an approved policy for the distribution of samples? | | --- | | |  | n/a | | --- | --- | | |
| | **If the entire sample will NOT be used during the course of this research study:** | | --- | |
| | Will the remaining tissue be discarded? If not what will be done with the remaining sample after study completion and how long will the sample be kept? | | --- | | |  | Yes | | --- | --- | | |
| | Will samples be made available to the research subject (or his/her medical doctor) for other testing? | | --- | | |  | No | | --- | --- | | |
| | **If a subject withdraws from the study:** | | --- | |
| | Will subject have the option to get the remaining portion of their sample back? | | --- | | |  | No | | --- | --- | | |
| | Will samples be destroyed? If not, will they be kept anonymously? What will happen to the sample if the subject revokes authorization? | | --- | | |  | Yes | | --- | --- | | |
| | Will data obtained from their sample be deleted? What will happen to the sample if the subject revokes authorization? | | --- | | |  | Samples will be destroyed if subjects revokes authorization. | | --- | --- | | |
| | Will study data or test results be recorded in the subject's medical records? | | --- | | |  | No | | --- | --- | | |
| | Will results of specific tests and/or results of the overall study be revealed to the research subject and or his/her doctor? | | --- | | |  | No | | --- | --- | | |
| | Please identify all third parties, including the subject's physician, to receive the test results. | | --- | | |  | Results of specific tests and/or results of the overall study will NOT be revealed to the research subject or his/her doctor. | | --- | --- | | |
|  |
| **SAMPLE:  Sputum** |
| | What is the purpose of the sample collection? | | --- | | |  | The presence of various drugs of abuse will be compared between the conventional 5-panel assay using urine samples versus outcomes obtained from new assays for saliva developed by Rice University scientists. All samples will de-identified then be sent to Rice University for testing and evaluation. These samples will not be used for genetic urposes and will not be banked. Samples will be destroyed immediately after analysis.  Method A- Aware Messenger (Kalypte, Inc.): This saliva collection kit consists of an oral fluid collection swab and a capped specimen collection tube that includes 1 mL of specimen extraction buffer.  Method B QuntiSAL (QuantiSAL Inc.): This saliva collection kit consists of an oral fluid collection swab with indicator window that turns blue when adequate sample has been collected) and a capped specimen collection/transport tube that includes 3 mL of specimen extraction buffer.  Method C- Expectorated unstimulated whole saliva (UWS): A member of the study staff will ask the participant to expectorate (spit) into a 50 mL centrifuge tube. The participant will provide as much saliva as possible, not to exceed 5 mL. | | --- | --- | | |
| | For blood draws, specify the amount drawn, in teaspoons, at each visit and across the course of the subjects entire participation time. | | --- | | |  | n/a | | --- | --- | | |
| | Is there the possibility that cell lines will be developed with this sample?No | | --- | |
| | Sample will be obtained from: | | --- | | |  | Other: participant | | --- | --- | | |
| | Will the sample be stripped of identifiers? | | --- | | |  | Yes | | --- | --- | | |
| | **If sample will be released outside the hospital:** | | --- | |
| | Will sample be released to anyone not listed as an investigator on the protocol? Will the information be identifiable, coded or de-identified? | | --- | | |  | samples for Rice University will be stripped of identifiers. | | --- | --- | | |
| | Will sample material be sold or transferred to any third parties? Will the information be de-identified? | | --- | | |  | no | | --- | --- | | |
| | **If sample will be banked for future use:** | | --- | |
| | Where will the sample be banked and for how long? | | --- | | |  | n/a | | --- | --- | | |
| | Does the banking institution have an approved policy for the distribution of samples? | | --- | | |  | n/a | | --- | --- | | |
| | **If the entire sample will NOT be used during the course of this research study:** | | --- | |
| | Will the remaining tissue be discarded? If not what will be done with the remaining sample after study completion and how long will the sample be kept? | | --- | | |  | Samples will be destroyed after analysis. | | --- | --- | | |
| | Will samples be made available to the research subject (or his/her medical doctor) for other testing? | | --- | | |  | No | | --- | --- | | |
| | **If a subject withdraws from the study:** | | --- | |
| | Will subject have the option to get the remaining portion of their sample back? | | --- | | |  | No | | --- | --- | | |
| | Will samples be destroyed? If not, will they be kept anonymously? What will happen to the sample if the subject revokes authorization? | | --- | | |  | Samples will be destroyed if subject withdraws consent. | | --- | --- | | |
| | Will data obtained from their sample be deleted? What will happen to the sample if the subject revokes authorization? | | --- | | |  | Data will not be deleted because all samples had been stripped of identifiers. | | --- | --- | | |
| | Will study data or test results be recorded in the subject's medical records? | | --- | | |  | No | | --- | --- | | |
| | Will results of specific tests and/or results of the overall study be revealed to the research subject and or his/her doctor? | | --- | | |  | no | | --- | --- | | |
| | Please identify all third parties, including the subject's physician, to receive the test results. | | --- | | |  | none | | --- | --- | | |
|  |
| **SAMPLE:  Urine** |
| | What is the purpose of the sample collection? | | --- | | |  | Urine toxicology for marijuana, opiates, cocaine, and amphetamines will be monitored during study visits, as through a qualitative urine test performed on site. Qualitative tests for cocaine are expected to be positive after the intravenous infusion sessions. Qualitative urine testing can produce false-positive results after exposure to medications or dietary substances. If qualitative urine testing indicates the presence of a drug of abuse other than cocaine following intravenous infusion sessions, the sample may be sent to an analytical laboratory to verify the result. An FDA approved rapid-result urine pregnancy test will be used (i.e. dipstick test). Subjects will be asked to sign a release of information form for study personnel to access medical records to obtain information regarding to outcome of a pregnancy that occurred during the study.   The presence of various drugs of abuse will also be compared between the conventional 5-panel assay using urine samples versus outcomes obtained from new assays for urine and saliva developed by Rice University scientists. 3cc of urine will be collected for these analyses. The samples will de-identified then be sent to Rice University for testing and evaluation. These samples will not be used for genetic purposes and will not be banked. Samples will be destroyed immediately after analysis. | | --- | --- | | |
| | For blood draws, specify the amount drawn, in teaspoons, at each visit and across the course of the subjects entire participation time. | | --- | | |  | n/a | | --- | --- | | |
| | Is there the possibility that cell lines will be developed with this sample?No | | --- | |
| | Sample will be obtained from: | | --- | | |  | Other: participants | | --- | --- | | |
| | Will the sample be stripped of identifiers? | | --- | | |  | Yes | | --- | --- | | |
| | **If sample will be released outside the hospital:** | | --- | |
| | Will sample be released to anyone not listed as an investigator on the protocol? Will the information be identifiable, coded or de-identified? | | --- | | |  | All samples for Rice University will be stripped of identifiers. | | --- | --- | | |
| | Will sample material be sold or transferred to any third parties? Will the information be de-identified? | | --- | | |  | No | | --- | --- | | |
| | **If sample will be banked for future use:** | | --- | |
| | Where will the sample be banked and for how long? | | --- | | |  | N/a | | --- | --- | | |
| | Does the banking institution have an approved policy for the distribution of samples? | | --- | | |  | n/a | | --- | --- | | |
| | **If the entire sample will NOT be used during the course of this research study:** | | --- | |
| | Will the remaining tissue be discarded? If not what will be done with the remaining sample after study completion and how long will the sample be kept? | | --- | | |  | Samples will be destroyed after analysis. | | --- | --- | | |
| | Will samples be made available to the research subject (or his/her medical doctor) for other testing? | | --- | | |  | No | | --- | --- | | |
| | **If a subject withdraws from the study:** | | --- | |
| | Will subject have the option to get the remaining portion of their sample back? | | --- | | |  | No | | --- | --- | | |
| | Will samples be destroyed? If not, will they be kept anonymously? What will happen to the sample if the subject revokes authorization? | | --- | | |  | Samples will be destroyed. | | --- | --- | | |
| | Will data obtained from their sample be deleted? What will happen to the sample if the subject revokes authorization? | | --- | | |  | Data obtained will be destroyed if subject revokes authorization | | --- | --- | | |
| | Will study data or test results be recorded in the subject's medical records? | | --- | | |  | No | | --- | --- | | |
| | Will results of specific tests and/or results of the overall study be revealed to the research subject and or his/her doctor? | | --- | | |  | No | | --- | --- | | |
| | Please identify all third parties, including the subject's physician, to receive the test results. | | --- | | |  | none | | --- | --- | | |
|  |
| Section O:  Drug Studies |
| | Is this study placebo-controlled? | | --- | | |  | Yes | | --- | --- | | |
| | Does the research involve a drug or biologic (including radioactive drugs) that is not approved by the FDA? | | --- | | |  | Yes | | --- | --- | | |
| | Will the research involve a radioactive drug that is not approved by the FDA? | | --- | | |  | No | | --- | --- | | |
| | IND Number: | | --- | | |  | 106,280 | | --- | --- | | |
|  |
| Section P:  Device Studies |
| | Does this research study involve the use of ANY device? | | --- | | |  | No | | --- | --- | | |
|  |
| [**Section Q:  Consent Form(s)**](javascript:openCF(235092, 0, 1)) |
| | Standard Consent Form | | --- | |
|  |
| **Section R:  Advertisements** |
| ADVERTISEMENT:  Newspaper |
| | Exact language of Advertisement: | | --- | | |  | Please see the attached two flyers. We would like to use the flyers on the internet, newspapers and bulletin boards. | | --- | --- | | |
| ADVERTISEMENT:  Radio |
| | Exact language of Advertisement: | | --- | | |  | Do you use cocaine or crystal meth? Do you know someone who does? Baylor College of Medicine’s Stimulant Addiction Research Program offers free comprehensive research opportunities for individuals who are not looking for treatment at this time. These research studies investigate potential treatment methods to treat cocaine and crystal addiction. Studies involve administration of either an experimental drug or placebo. Dr. Richard De La Garza of Baylor College of Medicine’s Department of Psychiatry and Behavioral Sciences is conducting several research studies offering complete medical exams and payment for research volunteers who are currently using cocaine and crystal meth. Call (877-228-5777) for more information. If eligible, cocaine or crystal meth study participants will be asked to stay several days in the hospital. If you or someone you know is interested in learning more about research opportunities for people who are using cocaine or crystal meth, please call Baylor College of Medicine’s Stimulant Addiction Research Program at (877-228-5777). Once again, that number is (877-228-5777). | | --- | --- | | |
| ADVERTISEMENT:  Other: Business Cards |
| | Exact language of Advertisement: | | --- | | |  | Are you currently using crystal methamphetamine or cocaine and not seeking treatment at this time? You may be eligible for a research study investigating potential treatment methods for stimulant addiction. Participants will receive some cash and/or gift certificates as compensation. Call us at 877 228-5777 for more information. | | --- | --- | | |
| ADVERTISEMENT:  BCM Clinical Trials Website |
| | Exact language of Advertisement: | | --- | | |  | We are currently looking for people who use cocaine for a 28 day, inpatient research study. The purpose of this study is to find out if doxazosin can reduce cocaine use and determine safety and effects of doxazosin when used together with cocaine. Participants will receive either placebo or doxazosin and a limited amount of cocaine or placebo, IV. They will have to answer questions about personal and family psychiatric history, including alcohol and drug abuse. Participants will be compensated for participating.   If interested, please call Jin Yoon, PhD at 713-791-1414 ext. 4153 for more information. All calls will be confidential. If you are seeking treatment, you should not participate in this study and the investigators can refer you to organizations that offer treatment for cocaine addiction. | | --- | --- | | |
|  |
